# Safety and Feasibility of Surgery for Oropharyngeal Cancers During the SARS-CoV-2-Pandemic

**DOI:** 10.3389/fonc.2021.651123

**Published:** 2021-03-24

**Authors:** Philippe Gorphe, Bruno Grandbastien, Andreas Dietz, Umamaheswar Duvvuri, Robert L. Ferris, Wojciech Golusinski, Floyd Christopher Holsinger, Sefik Hosal, George Lawson, Hisham Mehanna, Vinidh Paleri, Richard Shaw, Giovanni Succo, C. René Leemans, Christian Simon

**Affiliations:** ^1^Department of Head and Neck Oncology, Gustave Roussy, University Paris-Saclay, Villejuif, France; ^2^Department of Hospital Preventive Medicine, CHUV, University of Lausanne, Lausanne, Switzerland; ^3^Department of Otolaryngology, Head and Neck Surgery, University Leipzig, Leipzig, Germany; ^4^Department of Otolaryngology, University of Pittsburgh Medical Center, Pittsburgh, PA, United States; ^5^Department of Otolaryngology, University of Pittsburgh Medical Center, Pittsburgh, PA, United States; ^6^Department of Head and Neck Surgery, Poznan University of Medical Sciences, The Greater Poland Cancer Centre, Poznan, Poland; ^7^Division of Head and Neck Surgery, Department of Otolaryngology, Stanford University, Palo Alto, CA, United States; ^8^Department of Otolaryngology-Head and Neck Surgery, Atilim University Faculty of Medicine, Ankara, Turkey; ^9^ENT and Head and Neck Surgery Department, CHU UCL Namur- Site Godinne, Namur, Belgium; ^10^Institute for Head and Neck Studies and Education, University of Birmingham, Birmingham, United Kingdom; ^11^Head and Neck Unit, The Royal Marsden Hospitals NHS Foundation Trust, London, United Kingdom; ^12^Liverpool Head & Neck Centre, Aintree University Hospital, Liverpool, United Kingdom; ^13^Department of Molecular & Clinical Cancer Medicine, University of Liverpool, Liverpool, United Kingdom; ^14^Department of Oncology, University of Turin, Turin, Italy; ^15^Head and Neck Oncology Unit, Candiolo Cancer Institute - FPO IRCCS, Candiolo, Italy; ^16^Department of Otolaryngology-Head and Neck Surgery, Amsterdam University Medical Centres, Cancer Center, VU University, Amsterdam, Netherlands; ^17^Department of Otolaryngology – Head and Neck Surgery, CHUV, University of Lausanne, Lausanne, Switzerland

**Keywords:** oropahryngeal cancer, transoral robotic, head and neck cancer, SARS–CoV−2, COVID-19, head and neck surgery

## Introduction

At the end of December 2019, Chinese public health authorities reported several cases of acute respiratory syndrome in Wuhan City, Hubei province in China (1). Since then, SARS-CoV-2, the cause of coronavirus disease 2019 (COVID-19), has spread across the globe, leading to the World Health Organization declaring a Public Health Emergency of international concern on 30th January 2020, declaring a pandemic (https://www.who.int/docs/default-source/coronaviruse/situation-reports/20200407-sitrep-78-covid-19.pdf?sfvrsn=bc43e1b_2). After a first wave between March and June in Europe followed by a gradual reduction of daily infections a second more significant wave emerged with daily case rates peaking in the beginning of November. Although rates reduced somewhat since, they remain high and well above the rates of the first wave. As of end of December 2020, more than 79 million people had been infected and more than 1.7 million patients died worldwide (https://covid19.who.int/?gclid=EAIaIQobChMI76X8g4fx7QIVGqd3Ch2uyA92EAAYASAAEgJkxvD_BwE).

Early on in the pandemic, data from Wuhan emerged suggesting an increased mortality among surgical patients during the incubation period of a COVID 19 pneumonia. While a requirement of ICU care in 44% of patients was noted, the mortality amounted to 20.5% (2). Also, data emerged on the potential risk of infection of health care workers related to head and neck interventions (3). These concerns together with a mounting experience of resource constraints were taken into consideration, when consensus guidelines for head and neck cancer treatment were developed based on expert opinion and Delphi exercises for both radiation therapy (RT) and surgery (4, 5). In these guidelines it was strongly agreed on by experts that operations should be delayed until patients were negative on SARS-COV2 (repeat) tests. Also, for early-stage disease (i.e., T1, 2N0 oral cancers) a delay of up to 8 weeks with serial monitoring of the patient was found acceptable. To the contrary, for advanced-stage HNSCCs no delay was found to be acceptable. Early-stage oropharyngeal cancers were considered of lesser surgical priority and were ranking with that respect below advanced oral, laryngeal, and maxillary cancers, and also early-stage oral cancers. It was suggested that surgery for oropharyngeal cancer could be avoided as RT- and chemoradiation therapy (CRT) were considered to be alternative regimes that were comparably efficient in terms of cancer control with less risk for SARS-COV-2 related mortality (4).

We therefore thought it was critical to analyze the pertinent literature to determine the safety and feasibility of surgery, both open and by transoral approaches for oropharyngeal cancers during the ongoing pandemic.

## Risk Factors, Increased Mortality, and Severity of a SARS-CoV-2 Infection

Twenty-two percent of patients hospitalized with laboratory confirmed COVID-19 are critically ill as shown in a prospective observational cohort study of 1,150 patients. Factors associated with in-hospital mortality were found to be advanced age (aHR 1.31), chronic cardiac disease (aHR 1.76), chronic pulmonary disease (aHR 2.94), and certain laboratory parameters i.e., d-dimers and interleukin 6 (6).

Genome wide association studies (GWAS) helped to identify certain genetic factors associated with severe COVID-19. Among them the 3p21.31 gene cluster as a genetic susceptibility locus and the AB0 blood group system emerged to be associated with a higher risk for severe COVID-19 with an OR 1,45 of group A vs. an OR 0.65 of group 0, thus being protective (7). The severity of the SARS-Cov-2 infection has been reported to be associated with the RT-qPCR cycle threshold, which correlates with the estimated viral load (8, 9).

One of the most important factors impacting on the severity of a SARS-CoV2 infection is the presence of cancer. In a retrospective study on 232 COVID-19 patients with various types of cancers and 519 without cancer that were matched statistically, patients with cancer had an OR 3.61 to develop a severe form of the disease (WHO guidelines) (10).

Surgery in SARS-COV2 positive patients is plagued with a 24% mortality rate. This mortality is associated with age, male gender, ASA-status, presence of a cancer, undertaking of an emergency procedure, and undertaking of a major procedure (11).

## Risk Factors for Higher Morbidity of a SARS-CoV-2-Infection in Cancer Patients

A more severe SARS-CoV2 infection in cancer patients is associated with age, ECOG-status, cancer stage, and the application of targeted/immunotherapy (10).

As an independent risk factor for death a positive smoking history emerged from a register of 200 patients with thoracic malignancies (12). Other factors associated with increased 30 days mortality in a cancer population with a SARS-CoV2 infection were found to be increased age, male gender, smoking status, number of comorbidities, ECOG performance status 2 or higher, active cancer, receipt of azithromycin plus hydroxychloroquine (13).

Looking at various treatment modalities for cancers and contributing risks a retrospective multi-center study on 205 cancer patients with laboratory-proven SARS-CoV2 infections revealed that receiving chemotherapy within 4 weeks before onset of symptoms was an independent risk factor for death during admission to the hospital (14).

However, within the UK Coronavirus Cancer Monitoring Project (UKCCMP) it was demonstrated that after adjusting for age, gender, and comorbidities, chemotherapy had NO effect on mortality, neither had immunotherapy, hormonal therapy, targeted therapy, or radiotherapy all within the past 4 weeks (15).

## Treatment Considerations for Oropharyngeal Cancer (OPC) During the SARS-CoV-2 Pandemic

The treatment of OPCs should under normal circumstances not be delayed. A delay of 3 months would create a reduction of net survival by 16.8% in the age group between 50 and 59 and even 18.3% in the age group between 60 and 69 (16). Patients usually undergoing surgery for OPCs are often early-stage cancer patients and/or patients with early T-stages. Early stage HNSCC is particularly sensitive to a delay of treatment in terms of mortality risk as is OPC in comparison to oral cavity cancer (17).

Taken the risk of additional mortality from a SARS-COV2 infection into account, older patients rather not benefit from an immediate treatment of their OPCs. In fact, in case of a nosocomial SARS-CoV2 infection rate of 5% patients above an age of 60 have an overall higher mortality, if immediately taken for a treatment vs. waiting for 2 months, until the infection rate reduces (16).

Typically, early-stage OPCs are surgically removed using transoral robotic surgery (TORS) or transoral laser microsurgery (TLM). Both are endoscopic surgical techniques. Alternative treatments for oropharyngeal cancers consist of radiotherapy with or without cisplatin. Treatment usually depends on patient and institutional factors. Radiation therapy and chemotherapy seem not to increase the risk of mortality of cancer patients with a SARS-CoV2 infection and thus are reasonable treatment options (15). Surgery however may confer a significant risk of 30-days mortality in the range of 24% in case of a SARS-CoV2-infection, if extended surgery is performed and done in an emergency setting (11). However, a prospective international cohort study comprising 1,137 patients treated with surgery for HNSCCs during the first wave SARS-CoV2 pandemic demonstrated a 30-day mortality of only 1, 2%. In this cohort the rate of SARS-CoV2 infections was only 3% suggesting that head and neck surgery can be undertaken safely during the pandemic as a consequence of preoperative testing and proper safety precautions in the hospital (18). In a study at a tertiary care center in NYC including 11,540 patients prior undergoing surgery in an area of low COVID19 community rates, only 4,3% were found positive during the peak of the first wave declining to 0,3% (19). Finally, COVID-19-free “hubs” created to deliver cancer surgery during the pandemic have shown that cancer surgery after screening is a safe practice; in an international multicenter cohort study comprising 9,171 patients, COVID-19-free surgical pathways were shown to reduce the pulmonary complication rate from 4.9 to 2.2% consistent with rates seen in SARS-COV2 negative patients (20).

There remain reasonable concerns about the possible contamination of health care workers during rare head and neck surgery in SARS-COV2 -positive patients. Treating 29 of such patients out of 1,137 with head and neck cancers, health care workers developed COVID-19 in 24% of cases, which was significantly elevated (18). Comparing surgical techniques in terms of droplet counts known to greatly contribute to the transmission of SARS-COV2 during different types of procedures, the highest counts were found during osteotomies and no droplets were identified during i.e., TORS (21).

## Discussion

Surgery for oropharyngeal cancers during the pandemic seems feasible and safe. Delays should be in general avoided in particular for early-stage disease. An exception constitutes advanced age and high nosocomial SARS-COV2 infection rates, which should prompt a delay of treatment. SARS-CoV2 screening prior surgery and sanitary precautions taken by hospitals seem sufficient to maintain an acceptably low complication rate and mortality. TORS may confer an advantage as being a technique with less droplet distribution than open surgical techniques.

## Author Contributions

PG, BG, CL, and CS designed the study and collected the data. PG, BG, CL, CS, HM, VP, and RS interpreted the data. CS supervised the project. All authors drafted the manuscript and contributed to the article and approved the submitted version.

## Conflict of Interest

The authors declare that the research was conducted in the absence of any commercial or financial relationships that could be construed as a potential conflict of interest.
